# General Medicine and Therapeutics

**Published:** 1895-10

**Authors:** 


### GENERAL MEDICINE AND THERAPEUTICS.

EDITED BY DR. LUTHER B. GRANDY.

### THE PREVENTION OF DISEASE AMONG INFANTS.

The Board of Health of Newark, New Jersey, has issued the following bulletin on this subject:

This circular is advisory, and deals only with the prevention of disease. The care and treatment of a patient should be referred to a physician.

A large proportion of all who die are infants one year of age. By far the larger number of infants who die have been fed artificially. It has also been demonstrated that this mortality is chiefly caused by errors in infant feeding.

During the first year infants were designed to subsist on animal food (namely human milk); on this they usually thrive. When deprived of their natural food, the physician, the chemist, and the mother have been taxed to their uttermost to provide a suitable substitute.

The results of careful investigation and long experience have proved that fresh cow's milk, when it has been modified to correspond to woman's milk, and then, by some proper treatment, perserved from spoiling, is the best substitute food for the infant during its first year.

### ARTIFICIAL FEEDING.

The average stomach of a child at birth holds, when full, about two tablespoonfuls.

The increase in the size of the child's stomach is in proportion to its growth or weight.

The health and vigor of after life is undoubtedly laid in the first year, by proper feeding.

Proper infant feeding usually makes muscular children, with nerve force, not always fat ones.

When a food is substituted for woman's milk, it should contain only what nature designed, and in the same proportions.

Nature does not supply bread or crackers, or meat, or granulated sugar; and these should not be given to the infant.

Cow's milk, when properly prepared, furnishes a wholesome and sufficient diet for an infant, and supplies all it needs for robust health.

Fresh milk should constitute the principal article of food for the infant, even after weaning, and during the greater part of childhood.

No infant, under one year of age, can easily digest cow's milk until changed; it is weaker in some things, and stronger in others, than woman's milk.

Failures in artificial feeding are chiefly due to three causes. First: Overfeeding. Second: The use of food which is either too strong or too weak. Third: The use of food which is changing or has already spoiled.

The following receipts will change cow's milk into food mixtures suitable for healthy infants, up to one year :

### MODIFIED MILK FOR INFANT FEEDING.

MADE WITH ONE QUART OF BOTTLED COW'S MILK.

| *First* 6 *months.* | | *From* 6 *to* 9 *months.* | |
|---|---|---|---|
| The top milk (cream)..... | ½ pt. | The top milk (cream).. | 1 pt. |
| Boiled water.................. | 1 pt. | Boiled water............... | 1 pt. |
| Milk sugar.............. | .....700 grs. | Milk sugar................. | 900 grs. |

*From* 9 *months to* 1 *year.*

| | |
|---|---|
| Top milk................................ | .......1½ pts. |
| Boiled water.. | ................................½ pt. |
| White sugar ...... ......... ................ | 3 teaspoonfuls. |

Dissolve the sugar in hot water, add the cream, and divide in separate bottles, putting one feeding in each. Cork them with clean cotton.

One tablespoonful of lime water should be added to every gill of the food.

To preserve the food from spoiling, set the bottles filled and corked in boiling hot water for thirty minutes. A three-quart covered pail will answer.

### THE PROPER CARE OF MILK.

Milk is a delicate animal fluid, highly sensitive to exposure, and quickly spoils unless it receives great care.

Milk is spoiled by the bacteria which fall into it, and which set up fermentations due to their presence in it.

Vessels for holding milk should be made of earthenware, glass, or porcelain, and always be provided with covers.

In open vessels, milk should be counted unclean, for it is thus exposed to invisible droppings of dust.

All utensils designed for milk should first be scoured, then cleansed with soap, and rinsed with boiling water.

Bottles intended for milk should be cleansed with coarse sand, baking soda, and water; then rinsed and scalded.

Empty milk bottles should be properly cleansed; then filled with boiling water and allowed to stand until used.

Chemical poisons which germs cast off, and various germs of contagion, are the contaminations in milk most dreaded.

Heat and cold are preservatives when applied to milk; but extremes of either are injurious and destructive.

Heat is chiefly used to destroy the numerous germs which contaminate all milk, and which finally spoil it.

Cold is valuable because it retards the growth of germs, while applied to milk, but never any longer.

Milk should never be allowed to freeze, nor be subjected to more heat than necessary to sterilize it.

Milk is sterilized when it has been heated with steam or boiling water long enough to destroy the germs in it.

Milk is pasteurized which has been heated at 167 degrees Fahrenheit for twenty minutes and then cooled quickly.

Pasteurized milk is free from harmful germs, and has not been injured, as when completely sterilized or boiled.

Milk is best preserved when stored in small glass bottles, corked with cotton wool, and kept on ice.

When ice is not available, bottled milk should be immersed in cold water, which should be frequently changed.

When the separate feedings of milk are kept in small, closed bottles, the several portions are equally protected.

### THE HYGIENE OF THE NURSERY.

Regular habits, proper food, and long hours of sleep are necessary conditions to a healthy infant.

The three prime essentials in the nursery are fresh air, good food, and pure water.

Never put a bottle nipple into your mouth, and then into the baby's mouth; this will often prove dangerous.

Always hold a baby in your arms when feeding it, in about the same position as if nursing it.

Feeding in the night, after the third month, is both inconvenient and unnecessary; sleep at night is better than food.

Do not feed the baby because it cries; this may be due to pain, and it is hurtful to fill an infant's stomach at such a time.

Have a rule for feeding the baby and do not vary from it; without regularity the mother becomes a slave.

More infants' lives are taken by overfeeding than by starvation. Never liken an infant's digestion or diet to your own.

An infant's thirst is not quenched by milk; it needs clean water to drink with regularity.

Plain boiled water, given between feedings, will often aid the digestion and satisfy the child when restless.

Vomiting or diarrhea are indications that the child is either sick or approaching sickness, and probably needs a physician.

Cholera infantum would be of rare occurrence if proper attention was always given to the quality and quantity of the food.

A nursing mother who worries, or who is exhausted, or who indulges in excitement, may become a source of danger to her infant.

An infant is a creature of habit, and usually responds to the wish of the mother, if the mother has order in her will.

Rubber tubes, complicated nipples, and nursing bottles are dangerous and should never be used.

Light and loose clothing, frequent bathing, or cool sponging are necessities for the infant in hot weather.

Cleanliness, as applied to the body, the mouth, the food, the vessels, the clothing, the furniture, the floor, the carpets, the beds, and the atmosphere should be strictly observed.

---

IN the treatment of the milder grades of *gastric and gastro-intestinal catarrh*, Adjunct Professor Eshner prescribes *sodium phosphate* in doses ranging from fifteen grains to a dram. The larger doses are used when constipation is present; the smaller when merely a detergent and antacid effect is desired. The drug is directed to be taken in hot water, always before meals, sometimes only in the morning, at other times thrice daily. The concentration varies with the end to be attained, large quantities of water being used when the catarrhal condition is pronounced, minimal quantities when a laxative effect is desired.—*Philadelphia Polyclinic.*

